# Pulmonary Function after Weight Loss in Obese Women Undergoing Roux-en-Y Gastric Bypass: One-Year Followup

**DOI:** 10.1155/2013/796454

**Published:** 2013-02-19

**Authors:** Marcela Cangussu Barbalho-Moulim, Gustavo Peixoto Soares Miguel, Eli Maria Pazzianotto Forti, Flávio do Amaral Campos, Fabiana Sobral Peixoto-Souza, Dirceu Costa

**Affiliations:** ^1^Post Graduation Program of Physiotherapy of Federal University of São Carlos (UFSCar), Rod. Washington Luís, km 235, São Carlos, SP, Brazil; ^2^Meridional Hospital, Av. São Joao Batista, n 200, Cariacica, ES, Brazil; ^3^Post Graduation Program of Physiotherapy of Methodist University of Piracicaba (UNIMEP), Rod. do Açúcar, km 156, Piracicaba, SP, Brazil; ^4^Post Graduation Program of Physiotherapy of Nove de Julho University (UNINOVE), Av. Dr. Adolpho Pinto, n 109, Barra Funda, São Paulo, SP, Brazil

## Abstract

*Introduction*. Obesity is a condition that causes damage to the respiratory function. However, studies have demonstrated that weight loss due to bariatric surgery has resulted in a huge improvement on some lung volumes, but controversy still persists regarding the behavior of the respiratory muscle strength and IRV (inspiratory reserve volume). *Objective*. To evaluate the effect of weight loss, after 1 year of the Roux-en-Y gastric bypass surgery (RYGB), on the lung volumes and the respiratory muscle strength in obese women. *Methods*. 24 obese women candidates were recruited for RYGB. Lung volumes (spirometry) and respiratory muscle strength were evaluated in preoperative period and one year after surgery. *Results*. There was a significant increase in some lung volumes. However, when examining the components of the VC (vital capacity) separately, an increase in ERV (expiratory reserve volume) and reduction of IRV were observed. Moreover, a statistically significant reduction in the values of respiratory muscle strength was recorded: MIP (maximal inspiratory pressure) and MEP (maximal expiratory pressure). *Conclusion*. Weight loss induced by bariatric surgery provides an increase in some lung volumes of obese women, but reduction in IRV. Additionally, there was also a reduction in the respiratory muscle strength.

## 1. Introduction

Obesity is a condition that causes damage to the various body functions, such as cardiovascular, musculoskeletal, and metabolic functions amongst others [1]. The respiratory function is also affected by obesity, as excess fat deposited on the chest wall and the abdominal cavity affects the chest mechanics. This results in increased work of breathing, reduced lung volumes, dysfunction of the respiratory muscle, impairment in gas exchange, and reduced exercise tolerance [2–9].

A few studies have demonstrated that weight loss due to bariatric surgery has resulted in a huge improvement in some functions, such as decrease in hemoglobin and hematocrit [10], decreased heart rate and oxygen consumption [10], and reduction of insulin resistance [11]. In addition, especially improved lung function with increased forced vital capacity (FVC) [3, 12, 13] and forced expiratory volume in one second (FEV_1_), improved alveolar-capillary diffusion capacity [10] and improvement in gas exchange [12, 13] have also been observed.

There is strong evidence supporting the increase in FVC and ERV (expiratory reserve volume) after weight loss [3, 12, 13]. However, controversy still persists regarding the behavior of the respiratory muscle strength and IRV (inspiratory reserve volume) after weight loss caused by the Roux-en-Y gastric bypass surgery.

The objective of the present study was to evaluate the effect of weight loss, after 1 year of the Roux-en-Y gastric bypass surgery (RYGB), on the lung volumes and the respiratory muscle strength in obese women. 

## 2. Methods and Procedures

### 2.1. Patients

Were recruited 24 obese women candidates for RYGB at the Meridional Hospital. Patients with body mass index (BMI) 35–50 kg/m^2^ were included if they met the minimal criteria for bariatric surgery proposed by the World Health Organization (WHO) report of 2000 [1]. The following were not included in the study: patients suffering from pulmonary diseases or those unable to carry out the pulmonary function tests adequately, smokers, patients who did not attend the reevaluation 1 year after surgery, and patients refusing to sign the Informed Consent Term. The present study was approved by the Meridional Hospital Ethics Committee (Protocol number 01/07).

### 2.2. Pulmonary Function Tests

The evaluation of the pulmonary function was carried out by conventional spirometry using a personal computer version of the NDD EasyOne Spirometer Model 2001 (Medizintechnik AG, Zurich, Switzerland). Parameters, such as volume, capacity, and flow of the lungs, were directly evaluated by using the slow vital capacity (SVC), the forced vital capacity (FVC), and the maximum voluntary ventilation (MVV) tests, with volunteers in a sitting position and a minimum of three repetitions, as recommended by the American Thoracic Society (ATS) and the European Respiratory Society (ERS) [14]. The obtained results were expressed in absolute values and as percentages of the predicted reference values for the Brazilian population [15]. The SVC test produced the following variables: vital capacity (VC), tidal volume (VT), inspiratory reserve volume (IRV), and expiratory reserve volume (ERV). The FVC test allowed the determination of the forced expiratory volume in 1 s (FEV_1_) and the FEV_1_/FVC ratio. The MVV, a variable that evaluates the respiratory endurance, was expressed in liters per minute and as a percentage of the predicted reference value for the Brazilian population [15].

The respiratory muscle strength was determined through the maximal static respiratory pressures measured during forced inspiration and expiration—maximal inspiratory pressure (MIP) and maximal expiratory pressure (MEP). The measurement was carried out using an aneroid manometer (Wika, Iperó, SP, Brazil), calibrated in centimeter H_2_O (±300 cm H_2_O), and equipped with a 2 mm hole to relieve the oral pressure. The procedure was carried out as described by ATS/ERS [16]. MIP and MEP were determined using the residual volume and the total lung capacity, respectively, with the subjects in a sitting position. The inspiratory and expiratory efforts were held for at least 1 second. Patients carried out at least three acceptable inspirations/expirations wearing a nose clip for determining the two reproducible inspirations/expirations. The highest values were used in the analysis. The MIP and MEP values were also expressed as percentages of the predicted values, according to the equation proposed by Neder et al. [17].

The patients were evaluated in the preoperative period, and one year after surgery, they were asked to return for a reevaluation of the pulmonary function tests, by the spirometry and respiratory muscle strength.

### 2.3. Statistical Analysis

The data collected were expressed as mean ± standard deviation and analyzed by the Shapiro-Wilk test. After verifying the normal distribution of the variables, the paired *t*-test was used to compare the preoperative and 1-year postoperative results.

The Pearson correlation was used to correlate these variables: W/H ratio and ERV; IRV and MIP.

The sample size had an 80% power at the 5% level of significance with MEP as the main variable.

## 3. Results

The characteristics of the patients, such as age, BMI, weight, and W/H ratio, are shown in Table 1. There was a significant reduction in the values of weight, BMI, and W/H ratio 1 year after the surgery. However, these results were already expected. The BMI value returned to normal in 11 patients; in the other 11 patients, it lowered to the range of overweight (25–30 kg/m^2^), and only 2 patients remained obese, despite significant reduction in the BMI one year after surgery. Before surgery, 12 patients had hypertension, 7 had dyslipidemia, and 8 had diabetes. One year after the surgery, 6 patients continued with hypertension and 2 with dyslipidemia. However, the diseases were less severe than in the preoperative period.

On analyzing the variables that measure lung volumes, it was observed that there was a significant increase in the VC, FVC, and FEV_1_. However, when examining the components of the VC separately, an increase in ERV and reduction of IRV, keeping the VT unchanged 1 year after surgery, was observed. Furthermore, respiratory endurance assessed by MVV also increased after weight loss (Table 2).

Moreover, in assessing respiratory muscle strength, a reduction statistically significant in the values of MIP and MEP was recorded (Table 3).

**Figure 1:**
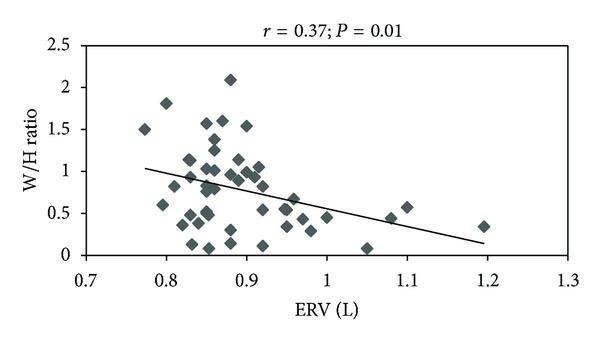
Correlation between W/H ratio and ERV (expiratory reserve volume). The data shown refers to the preoperative and postoperative values from 24 women patients.

**Figure 2:**
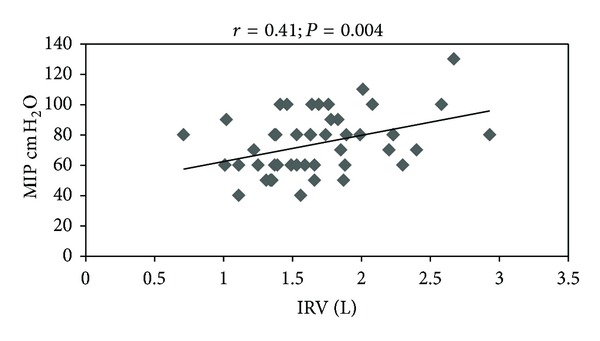
Correlation between MIP (maximal inspiratory pressure) and IRV (inspiratory reserve volume). The data shown refers to the preoperative and postoperative values from 24 women patients.

There was a significant negative correlation between W/H ratio and ERV (*r* = −0.37; *P* = 0.01) (Figure 1) and a significant positive correlation between MIP and IRV (*r* = 0.41; *P* = 0.004) (Figure 2).

## 4. Discussion

Based on the obtained results, it was established that 1 year after the RYGB surgery, the patients showed a significant reduction in the measures of weight, BMI, and W/H ratio, especially changes in the lung function tests, such as spirometry and respiratory muscle strength.

Studies by some authors [9, 12, 18] have shown an improved lung function in patients evaluated after 1 year following weight loss induced by bariatric surgery, and others have attributed this improvement mainly to the reduction in the W/H ratio. El-Gamal et al. (2005) [19] found that the patients showed an improvement in dyspnea and a reduction in the respiratory drive after weight loss induced by bariatric surgery. In the present study, one year after surgery, the patients showed increased lung volumes and decreased the respiratory muscle strength.

With respect to the volumes, an increase in VC, FVC, and FEV_1_ could be observed after weight loss. Other authors have also found similar results in previous studies [9, 12, 18, 20–22]. However, in this study, a finding still not published and discussed in the literature was noted: the IRV reducing associated with an increase of ERV after weight loss.

The reduction of ERV is a major known change in the respiratory function caused by obesity. According to Koenig (2001) [3], this fact is attributed to the reduction of the diaphragm mobility in the chest. This is because the diaphragm is pressed upwards due to the expanded abdominal volume and W/H ratio of the obese individuals, which is a mechanical disadvantage for this muscle. Besides these detrimental mechanics aspects to the pulmonary function of obese individuals, Young et al. 2003 [23] also suggested that the reduction of the ERV could lead to an increase in areas of atelectasis. As a result, the ventilation/perfusion mismatch could be harmed, thereby leading to arterial hypoxemia in those individuals. The reduction of W/H ratio after weight loss may have contributed to improvement in chest mechanics and consequent increase in ERV, as demonstrated by the significant correlation between these variables (*r* = −0.37; *P* = 0.01) (Figure 1).

Weiner et al. (1998) [22] also revealed an increase of ERV after weight loss. However, there is no report related to the reduction in IRV. Costa et al. (2008) [24] compared the pulmonary function in the obese and nonobese subjects, and the authors found a higher IRV and lower ERV in the first and the opposite in the other one, with no significant changes in the VC values between the groups observed. According to the authors, this is due to the problems in the chest mechanics of obese individuals, which could have resulted in a compensatory increase in IRV by reducing the ERV caused by obesity, while retaining an unchanged CV. Thus, in the present study there was a tendency to return to the patterns of distribution of the lung volumes of the non-obese individuals in studied patients. Therefore, the obtained results suggested that the weight loss induced by bariatric surgery altered the chest mechanics, by a rearrangement of the volumetric lung compartments inside the rib cage, especially for abdominal decompression after weight loss of these obese patients.

El-Gamal et al. (2005) [19] evaluated obese patients in the preoperative and 1 year after bariatric surgery and found that the low value of ERV is related to increased of respiratory drive and dyspnea, with improvement in these parameters after weight loss. These results help to consolidate the hypothesis that obesity, by reducing ERV, leads to a respiratory overload (inspiratory mainly), verified by the increase in dyspnea and respiratory drive [19], leading to increased of IRV. And finally, after weight loss these changes are reversed.

Besides these changes of the chest mechanics due to weight loss, the reduction of inspiratory muscle strength (MIP) may also have contributed to the reduction of IRV, since the inspiratory muscles are responsible for expanding the rib cage and promote entry of air into the lungs. This finding can be confirmed by the significant and positive correlation between these variables—MIP and IRV (*r* = 0.41; *P* = 0.004) (Figure 2).

The respiratory muscle strength is also an important variable that influences the lung function, and the available data in the literature are still few and controversial about this variable after weight loss. Dávila-Cervantes et al. (2004) [12] and Weiner et al. (1998) [22] also measured MIP and MEP after bariatric surgery and found an increase in these variables. Wadstrom et al. (1991) [25] found a reduction in the respiratory muscle strength after weight loss induced by bariatric surgery, in agreement with the results of the present study. One explanation for this finding could be the loss of lean body mass after bariatric surgery, as described by some authors [25–28].

The average weight loss with RYGB is 30% [29], which is similar to the results of the present study. However, this loss is not only fat of mass but also lean body mass. Stegen et al. [30] found a reduction in the lean body mass associated with a reduction of static and dynamic muscle strength, whereby the authors suggested that physical activity prevents the reduction of muscle strength after bariatric surgery. However, one limitation of the present study was that it did not evaluate the lean body mass of the patients.

Another hypothesis is that the reduced work of breathing in the obese individuals achieved with weight loss [19], no longer exerted “training” on the respiratory muscles of these individuals [24], thereby reducing the values of the respiratory muscle strength.

Despite a trend to decrease the maximal static respiratory pressures—MIP and MEP, there was an increase in MVV (a variable that evaluates the respiratory endurance). This fact can be justified as the weight loss promotes an improvement of the chest mechanics, increases the lung volumes [9, 22], and reduces the work of breathing [19].

Based on the obtained findings, it was concluded that weight loss induced by bariatric surgery provides an improvement in the ventilatory mechanics, as evidenced by the increase in lung volumes (ERV, VC, FVC, and FEV_1_) and respiratory endurance (MVV) of obese women. Furthermore, the reduction in IRV appears to show a trend distribution of static lung volumes in the pattern as seen in the nonobese patients. Additionally, there was also a reduction in the respiratory muscle strength, which could be caused by a loss of lean body mass and a reduction in the work of breathing after weight loss. However, further studies are warranted to confirm this hypothesis.

## Figures and Tables

**Table 1:** Values of age, weight, BMI, and W/H ratio.

Variable	Preoperative	Postoperative (1 year)	%Δ	*P*
Age (years)	42.41 ± 12.41	X		
Weight (kg)	103.64 ± 13.63	68.65 ± 10.24	−33.76	<0.0001
BMI (kg/m^2^)	40.17 ± 3.51	26.50 ± 3.22	−34.03	<0.0001
W/H ratio	0.93 ± 0.10	0.86 ± 0.04	−7.53	<0.0001

BMI: body mass index; W/H ratio: waist/hip ratio.

**Table 2:** Values of lung volumes (spirometry).

Variable	Preoperative	Postoperative (1 year)	%Δ	*P*
VC (L)	3.10 ± 0.65	3.21 ± 0.62	3.55	0.0356
% VC	92.29 ± 10.47	95.42 ± 12.44	3.39	0.0796
VT (L)	0.69 ± 0.28	0.70 ± 0.29	1.45	0.4357
IRV (L)	1.86 ± 0.39	1.48 ± 0.46	−20.43	<0.0001
ERV (L)	0.54 ± 0.33	1.02 ± 0.49	88.89	<0.0001
IC (L)	2.54 ± 0.45	2.17 ± 0.45	−14.57	<0.0001
FVC (L)	3.10 ± 0.70	3.33 ± 0.56	7.42	0.0009
% FVC	92.01 ± 13.07	98.88 ± 10.71	7.47	0.003
FEV_1_ (L)	2.49 ± 0.59	2.74 ± 0.48	10.04	<0.0001
% FEV_1_	88.79 ± 12.43	98.13 ± 10.58	10.52	0.0004
MVV (L)	101.59 ± 19.68	108.62 ± 14.25	6.92	0.0078
% MVV	86.50 ± 16.02	94.79 ± 22.22	9.58	0.0026

CV: vital capacity; VT: tidal volume; IRV: inspiratory reserve volume; ERV: expiratory reserve volume; IC: inspiratory capacity; FVC: forced vital capacity; FEV_1_: forced expiratory volume in one second; MVV: maximum voluntary ventilation.

**Table 3:** Values of respiratory muscle strength—MIP and MEP.

Variable	Preoperative	Postoperative (1 year)	%Δ	*P*
MIP cm H_2_O	78.75 ± 20.07	69.17 ± 18.86	−12.17	0.0183
% MIP	88.35 ± 23.61	77.45 ± 19.88	−12.33	
MEP cm H_2_O	92.08 ± 22.06	82.71 ± 18.71	−10.18	0.0078
% MEP	103.10 ± 27.98	93.77 ± 24.36	−9.05	

MIP: maximal inspiratory pressure; MEP: maximal expiratory pressure.

## References

[1] WHO (2000). Obesity: preventing and Managing the global epidemic. *Report of a WHO Consultation*.

[2] Faintuch J, Souza SA, Valezi AC, Sant’Anna AF, Gama-Rodrigues JJ (2004). Pulmonary function and aerobic capacity in asymptomatic bariatric candidates with very severe morbid obesity. *Revista do Hospital das Clinicas*.

[3] Koenig SM (2001). Pulmonary complications of obesity. *American Journal of the Medical Sciences*.

[4] Santamaria F, Montella S, Greco L (2011). Obesity duration is associated to pulmonary function impairment in obese subjects. *Obesity*.

[5] Lotti P, Gigliotti F, Tesi F (2005). Respiratory muscles and dyspnea in obese nonsmoking subjects. *Lung*.

[6] Rasslan Z, Junior RS, Stirbulov R, Fabbri RMA, Lima CAC (2004). Evaluation of pulmonary function in class I and II obesity. *Jornal Brasileiro de Pneumologia*.

[7] Jones RL, Nzekwu MMU (2006). The effects of body mass index on lung volumes. *Chest*.

[8] Steele RM, Finucane FM, Griffin SJ, Wareham NJ, Ekelund U (2009). Obesity is associated with altered lung function independently of physical activity and fitness. *Obesity*.

[9] Wei Y-F, Wu H-D (2012). Candidates for bariatric surgery: morbidly obese patients with pulmonary dysfunction. *Journal of Obesity*.

[10] Zavorsky GS, Kim DJ, Sylvestre JL, Christou NV (2008). Alveolar-membrane diffusing capacity improves in the morbidly obese after bariatric surgery. *Obesity Surgery*.

[11] Carvalho PS, Moreira CLCB, Barelli MC (2007). Cirurgia bariátrica cura Síndrome Metabólica?. *Arquivos Brasileiros de Endocrinologia & Metabologia*.

[12] Dávila-Cervantes A, Domínguez-Cherit G, Borunda D (2004). Impact of surgically-induced weight loss on respiratory function: a prospective analysis. *Obesity Surgery*.

[13] Zavorsky GS, Murias JM, Kim DJ, Gow J, Sylvestre JL, Christou NV (2007). Waist-to-hip ratio is associated with pulmonary gas exhange in the morbidly obese. *Chest*.

[14] Miller MR, Hankinson J, Burgos F (2005). Standardisation of spirometry. *European Respiratory Journal*.

[15] De Castro Pereira CA, Sato T, Rodrigues SC (2007). New reference values for forced spirometry in white adults in Brazil. *Jornal Brasileiro de Pneumologia*.

[16] American Thoracic Society/European Respiratory Society (2002). ATS/ERS Statement on respiratory muscle testing. *American Journal of Respiratory and Critical Care Medicine*.

[17] Neder JA, Andreoni S, Lerario MC, Nery LE (1999). Reference values for lung function tests. II. Maximal respiratory pressures and voluntary ventilation. *Brazilian Journal of Medical and Biological Research*.

[18] Martí-Valeri C, Sabaté A, Masdevall C, Dalmau A (2007). Improvement of associated respiratory problems in morbidly obese patients after open Roux-en-Y gastric bypass. *Obesity Surgery*.

[19] El-Gamal H, Khayat A, Shikora S, Unterborn JN (2005). Relationship of dyspnea to respiratory drive pulmonary function tests in obese patients before and after weight loss. *Chest*.

[20] Wadstrom C, Muller-Suur R, Backman L (1991). Influence of excessive weight loss on respiratory function. A study of obese pateints following gastroplasty. *Acta Chirurgica—European Journal of Surgery*.

[21] Nguyen NT, Hinojosa MW, Smith BR, Gray J, Varela E (2009). Improvement of restrictive and obstructive pulmonary mechanics following laparoscopic bariatric surgery. *Surgical Endoscopy and Other Interventional Techniques*.

[22] Weiner P, Waizman J, Weiner M, Rabner M, Magadle R, Zamir D (1998). Influence of excessive weight loss after gastroplasty for morbid obesity on respiratory muscle performance. *Thorax*.

[23] Young SS, Skeans SM, Austin T, Chapman RW (2003). The effects of body fat on pulmonary function and gas exchange in cynomolgus monkeys. *Pulmonary Pharmacology and Therapeutics*.

[24] Costa D, Barbalho MC, Miguel GPS, Forti EMP, Azevedo JLMC (2008). The impact of obesity on pulmonary function in adult women. *Clinics*.

[25] Wadstrom C, Muller-Suur R, Backman L (1991). Influence of excessive weight loss on respiratory function. A study of obese pateints following gastroplasty. *Acta Chirurgica—European Journal of Surgery*.

[26] Carey DG, Pliego GJ, Raymond RL (2006). Body composition and metabolic changes following bariatric surgery: effects on fat mass, lean mass and basal metabolic rate: six months to one-year follow-up. *Obesity Surgery*.

[27] Carey DG, Pliego GJ, Raymond RL, Skau KB (2006). Body composition and metabolic changes following bariatric surgery: effects on fat mass, lean mass and basal metabolic rate. *Obesity Surgery*.

[28] Olbers T, Björkman S, Lindroos A (2006). Body composition, dietary intake, and energy expenditure after laparoscopic Roux-en-Y gastric bypass and laparoscopic vertical banded gastroplasty: a randomized clinical trial. *Annals of Surgery*.

[29] Pataky Z, Carrard I, Golay A (2011). Psychological factors and weight loss in bariatric surgery. *Current Opinion in Gastroenterology*.

[30] Stegen S, Derave W, Calders P, Van Laethem C, Pattyn P (2011). Physical fitness in morbidly obese patients: effect of gastric bypass surgery and exercise training. *Obesity Surgery*.

